# Stimulation Modalities in Wearable Haptic Systems: Single-Mode Feedback to Multiphysics Actuation

**DOI:** 10.34133/research.1192

**Published:** 2026-03-31

**Authors:** Xu Guo, Raudel Avila, Ziqi Wu, Xinning Wang, Peixin Xu, Pengbin Ju, Qinzhe Yang, Pei Liu, Sherry Choi, Nikitsin Andrei, Zhaoqian Xie

**Affiliations:** ^1^ State Key Laboratory of Structural Analysis, Optimization and CAE Software for Industrial Equipment, Dalian University of Technology, Dalian 116024, China.; ^2^ Department of Engineering Mechanics, Dalian University of Technology, Dalian 116024, China.; ^3^ Department of Mechanical Engineering, Rice University, Houston, TX 77005, USA.; ^4^ Digital Health Institute, Rice University, Houston, TX 77005, USA.; ^5^ Rice Neuroengineering Initiative, Rice University, Houston, TX 77005, USA.; ^6^ DUT-BSU Joint Institute, Dalian University of Technology, Dalian 116024, China.; ^7^ Department of Bio and Nanomechanics, Belarusian State University, Minsk 220030, Belarus.

## Abstract

Wearable haptic systems enable natural and intuitive human–machine interaction, with growing relevance in immersive entertainment, physical rehabilitation, social communication, and personalized therapy. By delivering real-time tactile feedback via electrical, thermal, and mechanical stimulation, individually or in combination, these systems enhance user engagement and enable more dynamic and responsive digital interactions. This review presents a structured overview of the design principles, actuation mechanisms, and material architectures that define current wearable haptic technologies. We evaluate the strengths and limitations of major feedback modalities and highlight how system-level designs are tailored to specific use cases, from immersive gaming and training simulations to postoperative rehabilitation and emotion-sensitive communication. We also identify key challenges in miniaturization, multimodal integration, user comfort, and long-term wearability. The insights presented here aim to inform the development of next-generation haptic interfaces that are adaptive, scalable, and deployable in real-world applications.

## Introduction

Haptic feedback transmits physical sensations, including force, vibration, and temperature, directly to the skin, thereby providing a tactile channel for information exchange between users and digital systems. By engaging the sense of touch, haptic interfaces extend beyond the limitations of visual and auditory modalities, offering a powerful mechanism for interaction in applications such as postoperative rehabilitation, immersive gaming, hands-on educational simulation, and virtual reality environments that emulate texture and force [1]. Recent advancements in compact actuators, precision sensors, adaptive control algorithms, and flexible/stretchable materials have led to highly functional wearable haptic devices capable of delivering localized, low-latency stimulation with high spatial and temporal fidelity. These wearable haptic systems create a seamless interface between digital content and physical perception, enabling new modes of engagement in remote healthcare, assistive technologies, and multisensory virtual environments [2,3].

Wearable haptic devices operate in direct contact with the skin, necessitating thin, flexible, and conformal form factors for seamless integration with the human body. Meeting these constraints requires designs that balance key metrics including compactness, low mass, energy efficiency, comfort, and biocompatibility, while adapting to the distinct multiphysics demands of different actuation strategies. Pneumatic systems provide strong tactile forces but are hindered by latency and the size of the supporting infrastructure. Electrical stimulation offers compact, low-power operation but suffers from user-specific variability due to skin impedance and calibration sensitivity. Mechanical actuators respond rapidly and are easily miniaturized, although their output is constrained by limited displacement and frequency bandwidth. These trade-offs require integrated solutions that align materials, structural design, control/algorithmic logic, and wireless communication to achieve responsive, high-fidelity haptic interfaces optimized for wearability and real-world deployment [4,5].

Wearable haptic devices are rapidly advancing across domains such as healthcare, gaming, education, and industrial automation, each placing distinct demands on system integration and design. Clinical applications prioritize high-fidelity, low-latency feedback to support targeted therapies for motor rehabilitation and chronic pain. Immersive gaming emphasizes sustained, high-intensity stimuli to enhance user engagement, whereas educational and industrial contexts require robust, energy-efficient systems capable of delivering reliable feedback in remote instruction, simulation-based training, and human–machine collaboration. These design constraints, spanning response speed, stimulus strength, user comfort, and power consumption, require tailored integration strategies that align actuator capabilities with scenario-specific requirements [6–8].

Here, we present a comprehensive review of wearable haptic systems, focusing on structural architectures, actuation mechanisms, and emerging material platforms that enable conformal integration and precise tactile stimulation. We evaluate the functional advantages and trade-offs of mainstream feedback modalities across various representative application settings, including personalized rehabilitation and entertainment, as well as skill acquisition and automation. Finally, we outline the key engineering challenges that currently limit systems and discuss emerging trends that will shape the next generation of haptic interfaces for virtual reality, assistive care, and intelligent wearable technologies.

## Stimulation Modalities in Wearable Haptic Systems

Haptic perception is a complex sensory process that enables the detection of physical attributes such as shape, texture, temperature, and humidity through direct skin contact. It also allows the differentiation of variable mechanical cues, including pressure, shear, flexion, torsion, and vibration [9]. Haptic feedback technologies build on this biological foundation to artificially reproduce tactile sensations using actuators that simulate real-world stimuli. In human–computer interaction, haptic feedback enhances immersion, improves operational precision, and compensates for limitations in visual or auditory modalities, paving the way for seamless multisensory integration. Current research and industrial efforts focus on advancing the wearability of haptic systems by miniaturizing and reconfiguring conventional rigid components into soft, stretchable formats [10]. These design and optimization strategies increase user comfort and ensure stable, long-term interfacial strength and coupling between device and skin [5,11].

**Fig. 1.**
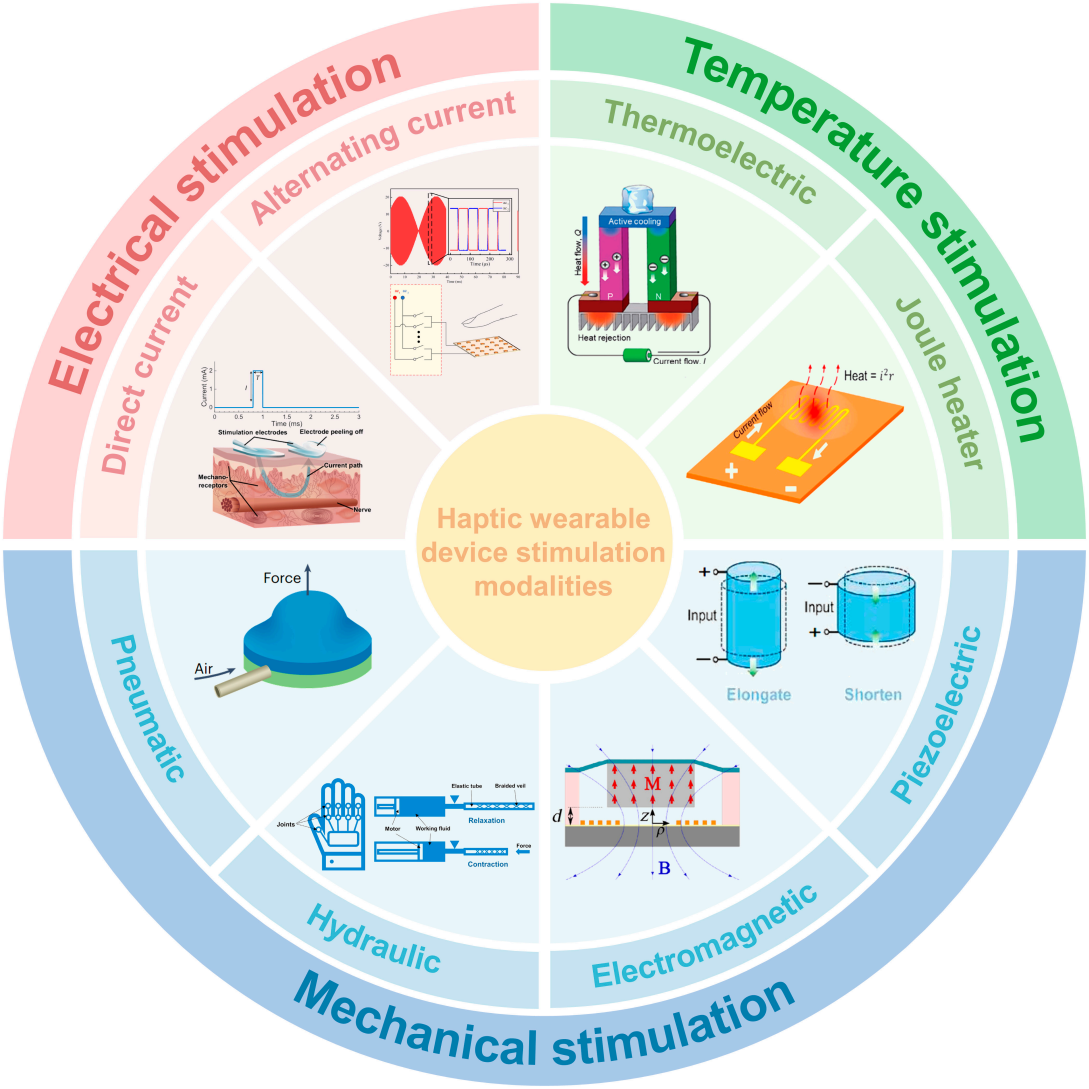
Representative stimulation modalities used in wearable haptic systems. Schematic overview of the 3 primary modes of tactile feedback: electrical stimulation, thermal stimulation, and mechanical stimulation. Each modality engages distinct physiological pathways and presents unique trade-offs in response time, spatial resolution, and integration complexity. Electrical stimulation includes direct current (DC) and alternating current (AC) approaches (reprinted with permission from [36], Copyright 2018, AAAS; [41], Copyright 2022, AAAS). Thermal stimulation includes thermoelectric and joule-heating approaches (CC-BY [118], Copyright 2022; reprinted with permission from [63], Copyright 2021, Elsevier). Mechanical stimulation includes pneumatic, hydraulic, electromagnetic, and piezoelectric approaches (reprinted with permission from [82], Copyright 2023, Wiley; [63], Copyright 2021, Elsevier; [88], Copyright 2015, Elsevier; [63], Copyright 2021, Elsevier).

As illustrated in Fig. 1, current wearable haptic systems primarily rely on 3 modes of tactile stimulation:

1. Electrical stimulation uses controlled currents to activate cutaneous mechanoreceptors and peripheral nerves, thereby eliciting haptic sensations. This modality operates on the principle of transcutaneous electrical nerve stimulation, in which applied current waveforms modulate the generation of action potentials in sensory nerves. Common approaches include direct current (DC), alternating current (AC), and user-specific waveform modulation [4,12,13].
2. Thermal stimulation replicates temperature sensations by actively modulating device surface temperature and local thermal diffusion, typically using joule heating or thermoelectric modules [14,15]. The underlying mechanism involves conductive and convective heat transfer between the stimulator and the skin, which engages cutaneous thermoreceptors through transient or steady-state thermal gradients.
3. Mechanical stimulation uses microscale actuators or dynamic structures to mimic pressure and vibratory cues, including pneumatic and hydraulic systems [16–18], as well as electromagnetic and piezoelectric actuators [19–22]. These systems generate tactile sensations by applying controlled force or displacement to the skin, thereby mechanically activating cutaneous mechanoreceptors, including Merkel cells, Meissner corpuscles, and Pacinian corpuscles.

To understand how these stimulation modalities interact with the human sensory system, it is necessary to consider the biological basis of tactile perception. Human skin contains specialized cutaneous receptors that respond to distinct physical stimuli with characteristic sensitivity, adaptation rates, and spatial resolution. Table 1 summarizes the key properties of these tactile receptors and provides a neurophysiological framework for evaluating the effectiveness of the haptic actuation technologies discussed in the following sections.

**Table 1.** Classification and characteristics of cutaneous tactile receptors

Receptor type	Adaptation	Typical depth (mm)	Frequency sensitivity (range; most sensitive band)	Density/spacing at fingertip	Primary stimulus
Merkel (SA I)	Slow	~0.5–1 [29]	≤100 Hz; most sensitive ~ 0.3–5 Hz [29]	~100 units/cm^2^ [29]	Static pressure
Meissner (FA I)	Fast	~3–4 [30]	~ 1–300 Hz; most sensitive ~ 5–50 Hz [29]	~ 150 units/cm^2^ [29]	Low-frequency vibration
Pacinian (FA II)	Fast	>10 [30]	~5–800 Hz; most sensitive ~30–500 Hz [29]	Sparse (large receptive fields) [29]	High-frequency vibration
Ruffini (SA II)	Slow	>7 [30]	~100–500 Hz (sensitive to stretch/strain) [29]	–	Stretching
Free nerve endings	–	–	–	–	Nociception, thermoreception
Krause end bulbs	Slow	–	~40–80 Hz [30]	–	Thermoreception

Each haptic stimulation modality presents distinct advantages and limitations, making it challenging for a single modality to deliver comprehensive haptic feedback or meet the multiphysics demands of complex and evolving virtual environments. To address this, recent efforts have focused on multimodal integration, combining complementary actuation strategies to achieve more nuanced and realistic haptic experiences. For example, hybrid systems that combine electrical and thermal stimulation have been shown to enhance sensory realism by coupling neurostimulatory and thermal effects to reproduce natural skin sensations, such as warmth or cooling accompanied by tactile feedback [23]. In addition, integrating mechanical actuation with human–machine interfaces (HMIs) improves the fidelity of interactive control [24]. The choice and combination of modalities are closely tied to specific application requirements. For example, in virtual reality, multimodal systems are often necessary to create immersive, lifelike interactions [25], whereas in medical rehabilitation, the focus is on precisely controlling electrical and mechanical stimuli to support sensory recovery [26]. Advances in algorithms and data-driven control have further improved the accuracy and adaptability of haptic feedback, enabling more intelligent and responsive systems for next-generation interactive applications [27–30].

## Electrical Interfaces for Tactile Feedback

Electro-haptics involves the application of electrical currents to targeted regions of the skin via conductive electrodes, stimulating local mechanoreceptors to initiate action potentials that propagate through the nerves and elicit tactile sensations in the brain. Foundational studies of electrically induced haptic perception date back to the 1940s [31]. Over decades of technological refinement and optimization, electro-haptic stimulation has evolved to selectively engage distinct mechanoreceptors by carefully tuning current parameters such as frequency, amplitude, and polarity. Single-neuron experiments have shown that Merkel receptors mediate pressure sensations, while Meissner corpuscles are responsible for vibration [32]. Kajimoto et al. [33] demonstrated that cathodal stimulation preferentially activates Merkel corpuscles, whereas anodal stimulation recruits Meissner corpuscles. Their work introduced the concept of “tactile primary colors,” referring to the reconstruction of complex haptic experiences through the selective activation of receptor subtypes.

**Fig. 2.**
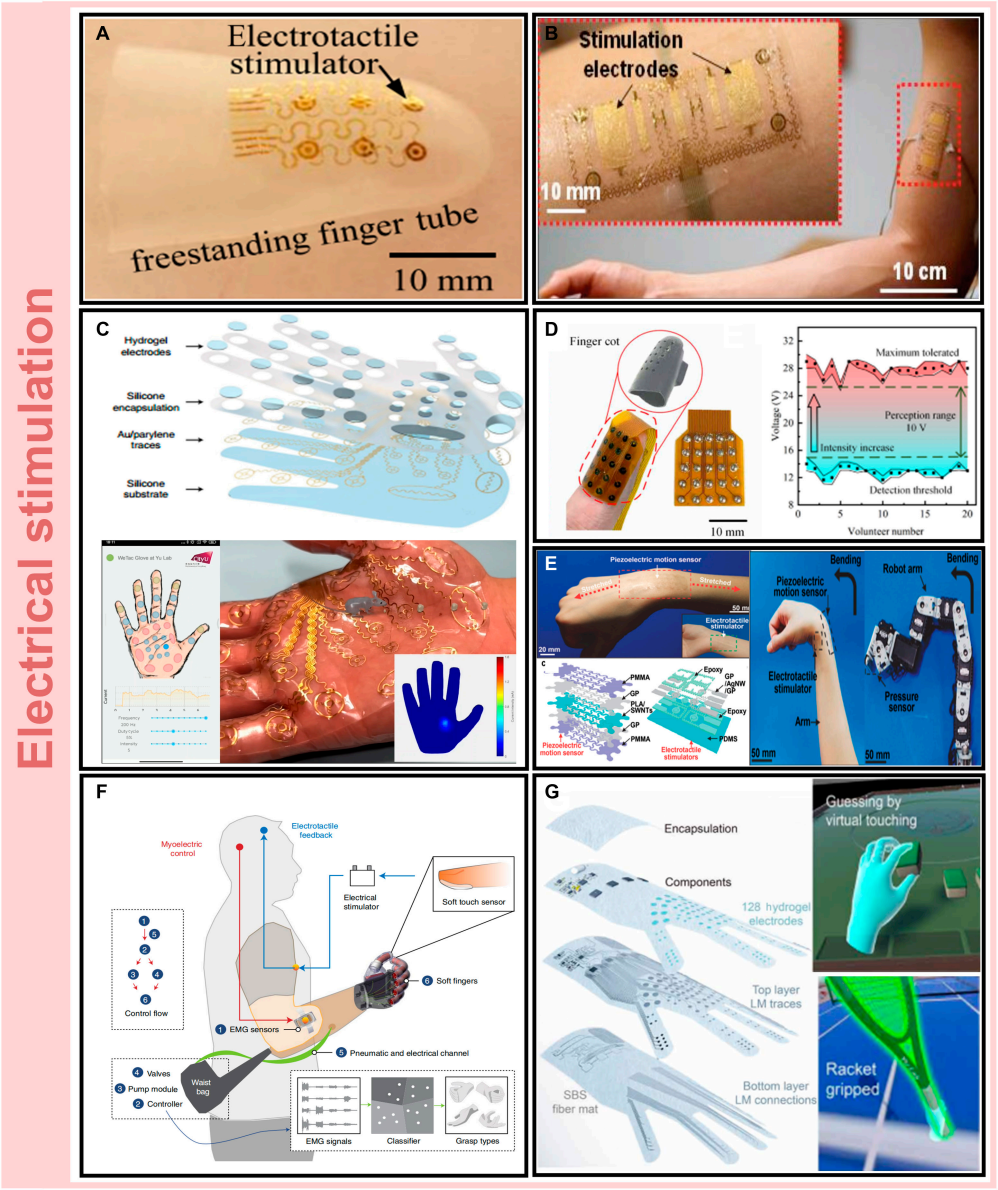
Representative wearable electro-haptic systems. (A) Ultrathin fingertip sleeve with electrotactile stimulation enabled by silicon nanomembrane diodes for circuit multiplexing. Copyright 2012, IOP [34]. (B) Multifunctional epidermal platform integrating electro-haptic output with electromyography, strain, and temperature sensing for motion management. Copyright 2016, Wiley [35]. (C) Personalized high-density wireless electrotactile system (WeTac) with dynamic stimulation threshold mapping. Copyright 2022, Springer Nature [40]. (D) High-frequency AC electro-haptic device enabling low-voltage operation and high-resolution feedback across 25 electrodes. Copyright 2022, AAAS [41]. (E) Transparent, stretchable system integrating electrotactile feedback and piezoelectric sensing for bidirectional human–robot interfaces. Copyright 2014, Wiley [42]. PMMA, polymethyl methacrylate; SWNTs, single-walled carbon nanotubes; AgNW, silver nanowire; PDMS, polydimethylsiloxane; GP, graphene; PLA, polylactic acid. (F) Neuroprosthetic hand with myoelectric control and real-time electrotactile feedback delivered to the residual limb. Copyright 2023, Springer Nature [26]. (G) Breathable, textile-based electro-haptic system with 128 stimulation pixels for extended virtual reality interaction. SBS, poly(styrene-block-butadiene-block-styrene); LM, liquid metal. Copyright 2024, AAAS [45]. Copyright as indicated; all reproduced with permission.

Electro-haptic interfaces form direct contact with the skin to stimulate mechanoreceptors, eliminating the need for traditional gel electrodes and enabling ultrathin, conformal designs. Recent research advances leverage nanomaterials for device miniaturization, serpentine interconnect wiring to accommodate large mechanical strain, and photolithography combined with transfer printing for microscale integration [34,35]. For example, Ying et al. [34] developed a 0.5-mm-thick silicone nanofingertip sleeve incorporating silicon nanomembrane diodes for signal multiplexing and applied electrotactile stimulation through 6 fingertip electrodes using a tubular inversion process (Fig. 2A). Xu et al. [35] introduced a 20-μm-thick multifunctional epidermal electronic system based on a water-soluble polyvinyl alcohol substrate, integrating electromyography (EMG), temperature, and strain sensors alongside electrotactile outputs (Fig. 2B). These mechanically ultrathin platforms offer exceptional comfort, stretchability, and utility in motion tracking and health monitoring. However, electrode–skin contact is often compromised by perspiration and movement, resulting in fluctuating impedance that concentrates current in localized regions, raising the risk of electric shock, discomfort, or thermal injury and posing major challenges for user safety and long-term wearability [36,37].

While subcutaneous microneedle electrodes can stabilize skin–electrode impedance [38,39], their invasive nature has motivated the development of noninvasive alternatives. Akhtar et al. [36] addressed this challenge by modeling the relationship between stimulation parameters and impedance under constant perceptual intensity and implemented a control system to dynamically adjust current amplitude and pulse duration. Electro-haptic interfaces also face variability in user-specific and site-specific sensitivity, limiting generalizability across applications. To overcome this, Yao et al. [40] introduced a soft, ultrathin, wireless electrotactile system (WeTac) designed for personalized stimulation (Fig. 2C). By mapping threshold distributions across users and body regions and optimizing frequency and duty cycle parameters, the wearable system enabled high-resolution stimulation using 32 electrodes, achieving a density of 0.279 pixels/cm^2^ on the palm. Lin et al. [41] further advanced this concept with a lightweight, wearable interface using amplitude-modulated high-frequency AC to reduce skin impedance. This approach lowered the stimulation threshold to 13 V, enabling safe, high-resolution feedback across 25 fingertip electrodes (Fig. 2D).

Electro-haptic systems, characterized by rapid response times and high spatial resolution, offer strong potential for HMIs [42,43], prosthetic feedback [26,44], and immersive virtual environments [45–47]. Lim et al. [42] developed a transparent, stretchable HMI platform incorporating electrotactile feedback (Fig. 2E). By analyzing the effects of electrode spacing, skin thickness, and stimulation frequency on detection thresholds, they enabled seamless bidirectional communication between human users and robotic systems. Gu et al. [26] engineered a neuroprosthetic hand equipped with electrotactile feedback, where pressure signals from soft capacitors at the prosthetic fingertips were relayed to electrodes on the residual limb (Fig. 2F). Yao et al. [45] further advanced wearable haptics with a fully integrated breathable haptic textile, engineered using an electrospun polymer substrate to improve breathability and minimize sweat interference. Featuring 128 stimulation pixels on the palm, the system enabled continuous, high-resolution feedback for virtual reality interactions (Fig. 2G).

Electro-haptic systems offer distinct advantages, including ultrathin form factors, high spatial resolution, and rapid temporal response. By tuning stimulation parameters, these interfaces can emulate complex tactile sensations such as pressure, vibration, and friction, positioning them as key enablers for next-generation human–machine interaction and virtual reality. However, unresolved challenges—such as unstable electrode–skin impedance, user-specific variability in perception thresholds, and safety concerns associated with high-voltage operation—continue to limit widespread adoption and long-term usability.

Table 2 summarizes representative wearable electro-haptic systems. In most studies, the skin–device interface is designed to be thin and compliant (e.g., using ultrathin patches, ring electrodes, or flexible printed circuits). Meanwhile, bulkier components for power and control are housed in compact driver modules placed away from high-mobility areas such as the fingers and hand, minimizing interference with natural movement [40,41]. The current is typically regulated with precision by controlling its amplitude, frequency, and duty cycle to ensure perceptible yet gentle stimulation of cutaneous nerves [41]. Monophasic pulses, which deliver current in a single direction, are commonly used for simple and low-cost stimulation [34,35,40,45]. Biphasic pulses achieve charge balance by AC direction, which effectively prevents charge accumulation in the tissue and thereby minimizes the risk of tissue damage [48–52]. Another method is amplitude-modulated high-frequency AC, which overcomes the insulation of the high-impedance stratum corneum, allowing the operating voltage to be reduced as low as 13 V, much lower than traditional electrotactile stimulators, which rely on high-voltage DC pulses (hundreds of volts), improving the safety during the electrotactile stimulation [37]. Each stimulation technique has distinct advantages, and the choice among them depends on the desired application. Maintaining stable electrode–skin impedance (e.g., with the aid of hydrogel interfaces) is crucial for consistent current delivery and for preventing sensation drift [40,45]. Therefore, the combination of precise current control and stable impedance forms the basis for safe stimulation, effectively reducing the risk of skin or nerve discomfort or injury.

**Table 2.** Comparative evaluation of electro-haptic systems

Type	Body location	Frequency	Thickness	The center-to-center distance between electrodes
Monophasic pulses [34,35,40,45]	Hand [40,45], fingertip [34], arm [35]	20 Hz [35], 25–500 Hz [40,45]	0.02–0.5 mm [34,35], 0.22–1 mm [40,45]	2–6 mm [34], 4.5–13 mm [40,45]
Biphasic pulses [48–52]	Wrist [48,51,52], sole of foot [49], torso [50]	50 Hz [49,50], 100 Hz [48], 2–1,000 Hz [51,52]	0.1–0.57 mm [49,51,52]	3–10 mm [48,51,52], >15 mm [49], 45 mm [50]
Amplitude-modulated high-frequency AC [41]	Fingertip	4–20 kHz	NR	~4 mm

NR, not reported.

A primary challenge for electro-haptic systems is to achieve high spatial resolution while maintaining wearing comfort and ensuring long-term biocompatibility. In general, achieving high resolution typically calls for reduced electrode spacing, which can raise local current density and increase mechanical stiffness, making stimulation feel sharper and less comfortable [36,37]. A practical path forward is to adopt a distributed island–bridge electrode layout that disperses mechanical stress, helping preserve user comfort even when reduced electrode spacing is needed for high resolution [34,35]. The contradiction between wearing comfort and long-term biocompatibility lies in the fact that achieving long-term stable skin adhesion and reliable contact often requires strong adhesion, an effective encapsulation layer, or a certain level of contact pressure, which compromises breathability and mechanical compliance. Under real wearing conditions such as exercise and sweating, the skin barrier becomes more susceptible to disruption, increasing the risk of discomfort and inflammation and potentially causing an imbalance in the local microbial environment [45]. To address the core contradiction between wearing comfort and long-term biocompatibility, a codesign strategy integrating the interface structure, materials, and functionality is required. Specifically, introducing bioinspired hydrogels at the electrode–skin interface, combined with breathable and compliant materials such as advanced functional textiles, can maintain reliable long-term electrical contact while preserving good breathability and mechanical compliance, thereby maintaining a skin-friendly local microenvironment [40,45]. Ultimately, to reconcile resolution, comfort, and long-term biocompatibility, a new generation of electro-haptic systems can be realized through the coordinated optimization of electrode layout to reduce local current density and mechanical stiffness [23], combined with the design of material properties to enhance breathability, compliance, and interfacial stability [40,45], alongside fine-tuning stimulation parameters to ensure safe thresholds and mitigate skin irritation [41].

## Thermal Interfaces for Tactile Feedback

Thermal haptic sensation arises from temperature changes at the skin surface and is detected by free nerve endings and specialized thermoreceptors such as Krause’s end bulbs. These receptors continuously monitor thermal exchange between the skin and external objects, transducing temperature gradients into neural signals that contribute to fine-grained thermal perception [23]. In virtual and augmented reality (VR/AR), temperature-based stimulation plays a critical role in enhancing realism by conveying the thermal properties of virtual objects, thereby deepening user immersion and sensory engagement [53–57].

**Fig. 3.**
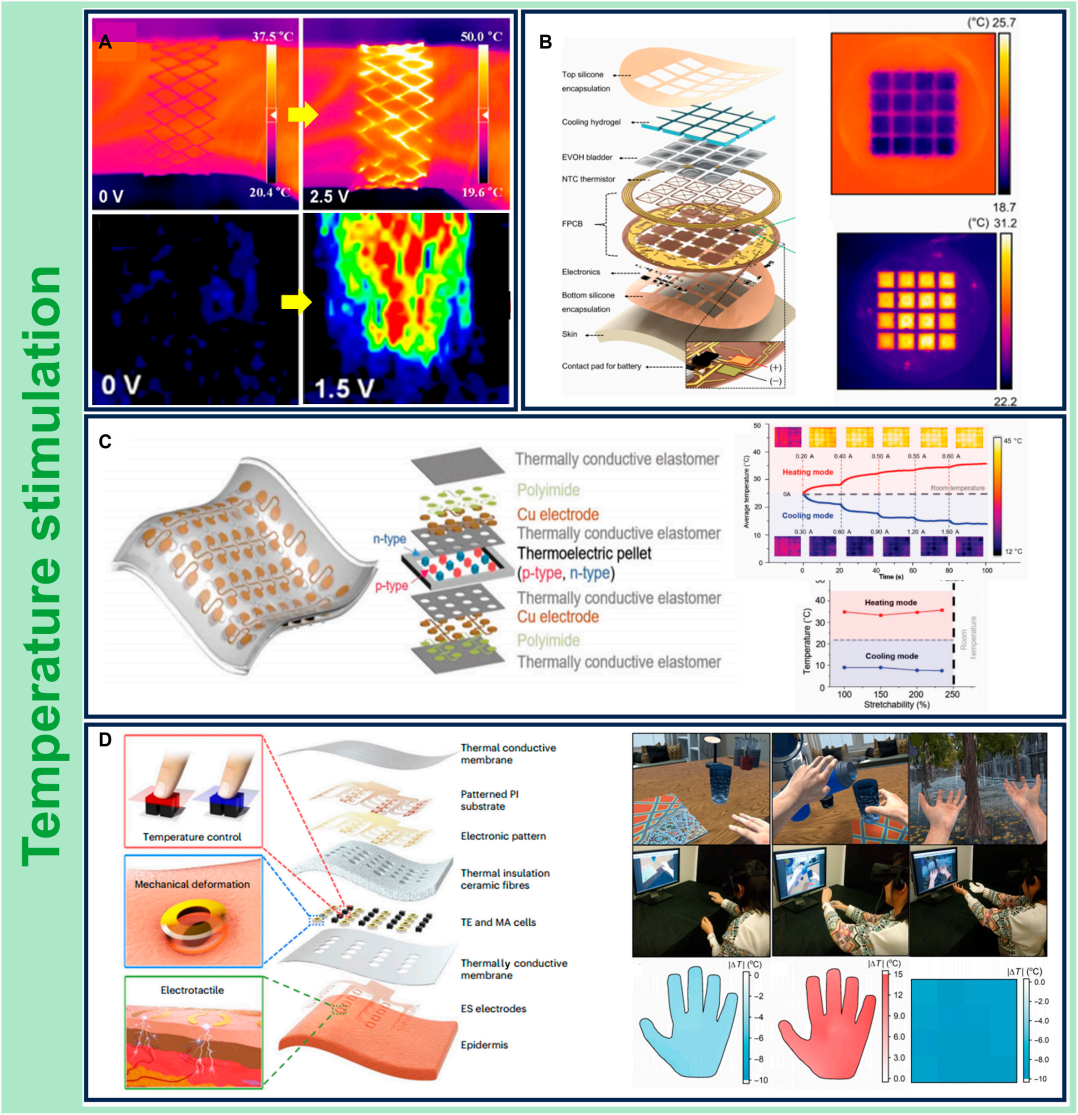
Representative wearable thermo-haptic systems. (A) Stretchable paper-cutting-based electrothermal device enabling rapid heating above 40 °C at low voltage with high mechanical durability. Copyright 2017, American Chemical Society [60]. (B) Skin-integrated platform combining resistive heating, passive cooling, and wireless closed-loop control for bidirectional temperature modulation over large epidermal areas. EVOH, ethylene-vinyl alcohol; NTC, negative temperature coefficient; FPCB, flexible printed circuit board. Copyright 2023, National Academy of Sciences [61]. (C) Skin-like thermo-haptic device (STH) offering reversible heating and cooling via a single thermoelectric interface with fingertip-level spatial precision. Copyright 2020, Wiley-VCH Verlag [14]. (D) Thermoelectric interface featuring a multilayer sandwich structure and embedded heat management for simulating temperature cues such as cold metal or warm fabric. TE, thermoelectric; MA, mechanical actuator; ES, electrotactile. Copyright 2023, Springer Nature [23]. Copyright as indicated; all reproduced with permission.

Realizing these perceptual effects in practice requires precise thermal actuation strategies integrated into skin-conformal platforms. Temperature stimulation in wearable haptic systems is primarily achieved through 2 mechanisms: joule heating and thermoelectric modulation. Joule-based thermal interfaces generate heat via electrical current passing through conductive elements, enabling localized skin heating for haptic simulation [15,58–62]. For example, Jang et al. [60] present a simple yet highly efficient way of producing high-performance stretchable heaters inspired by paper-cutting designs, using patterned conductive paper to fabricate a skin-conformal device (Fig. 3A). The system heats rapidly to over 40 °C at a low voltage of 1.2 V, with a thermal response time under 60 s. It retains functionality under 400% axial strain and remains stable over 1,000 mechanical cycles. When conformally applied to the wrist, the device conforms to skin during movement and provides consistent heat delivery, improving local blood flow and demonstrating utility for pain relief and joint rehabilitation.

Building on this wearable biointegrated approach, Park et al. [61] advanced thermal haptics further by integrating active cooling and closed-loop wireless control into a fully skin-interfaced platform that enables precise bidirectional thermal control using a passive cooling layer, an active thermal barrier, thin-film resistive heaters, and wireless electronics (Fig. 3B). The device supports closed-loop, spatiotemporal temperature regulation ranging from 27 to 40 °C within 7 s. Clinical applications span medical rehabilitation and remote care. In one experimental demonstration, the device maintained a stable back temperature of 32 °C when worn by a neonate, addressing impaired thermoregulation in newborns. Through cloud-based communication, the system also enables wireless transmission of thermal stimulation patterns in real time between users, mimicking the sensation of human warmth and physical presence, such as the thermal gradients generated from a hug.

While joule-heated interfaces offer rapid and stable heating, they often require external cooling components due to the absence of integrated microscale heat management and dissipation features, limiting their practicality for continuous use in wearables [63]. To overcome this limitation, thermoelectric haptic systems have emerged as a promising alternative, enabling bidirectional thermal control through the Peltier effect [64,65]. Unlike joule-heating devices, which can only generate heat, thermoelectric modules control current direction to induce either heating or cooling, offering greater versatility in temperature feedback [66]. Lee et al. [14] developed a skin-like thermo-haptic (STH) interface capable of delivering both hot and cold sensations through a single, highly soft, and stretchable form platform (Fig. 3C). The system uses feedback-controlled thermoelectric modules to enable real-time, precise temperature modulation with a maximum temperature differential of 20.7 K and a mechanical stretchability up to 230% in cooling mode. Critically, by reversing the applied voltage, the STH device can rapidly switch between heating and cooling, achieving temperature recovery rates more than 9 times faster than passive convection. When integrated into a motion-tracking glove, the system reproduces realistic thermal feedback, such as the sensation of touching a cold glass or a warm mug, with temperature errors under 1%. Moreover, an independent STH fingertip unit enables spatially distinct thermal cues, such as the thumb finger touching a cold drink while the index finger touches a warm drink, thus supporting refined, multipoint thermo-haptic feedback.

Further advancing the thermal fidelity and responsiveness of thermoelectric interfaces, Huang et al. [23] engineered a multilayered sandwich structure (Fig. 3D) that improves heat management and overall device performance. The device incorporates high thermal conductivity encapsulation materials and an embedded heat dissipation architecture to improve its efficiency. To minimize thermal backflow between layers, an adiabatic material was inserted between the 2 thermoelectric stacks. This stacking design enabled rapid thermal transitions, reaching a 12 °C increase in heating mode and a 7 °C decrease in cooling mode within 5 s. In virtual reality environments, the system accurately simulates the sensation of touching cold metal or warm fabric. Under transient, dynamic scenarios, such as a simulated rainstorm, it simulates both the cooling sensation and the haptic feedback of raindrops on the users’ skin by integrating temperature and mechanical impact cues.

Although thermoelectric haptic systems can deliver both heating and cooling stimuli, the cooling mode typically requires higher currents, which counterproductively generate considerable joule heat. This added thermal load increases overall power consumption and reduces the haptic device’s ability to achieve efficient cooling sensations [14,67]. To address these limitations, Ma et al. [68] developed a cooling device with high intrinsic thermodynamic efficiency using a flexible electrocaloric (EC) polymer film and an electrostatic actuation mechanism. The EC effect describes a reversible temperature change in dielectric materials induced by electric-field-driven modulation of dipole entropy. In this system, electrostatic forces rapidly shuttle the flexible EC polymer stack between a heat source and heat sink, enabling efficient heat exchange while minimizing parasitic energy loss. Despite these advantages, the cooling cycle requires periodic actuation to establish and break thermal contact with the EC layer, reducing energy efficiency and introducing additional complexity in fabrication and integration.

Table 3 compares the materials, dimensions, and response time of joule-heating and thermoelectric thermo-haptic systems. Joule-heating and thermoelectric thermo-haptic systems differ most clearly in energy efficiency, thermal safety, and cooling power requirements [69,70]. Joule heaters are energy-efficient for heating because electrical power is converted directly into heat (e.g., >40 °C at 1.2 V) [46], but they cannot actively cool and must rely on passive heat dissipation or additional components [60]. Thermoelectric devices provide bidirectional heating and cooling [14,69]. In practice, effective cooling often requires high operating currents, which cause parasitic joule heating and reduce cooling efficiency [63]. In addition, ensuring efficient thermal stimulation often requires optimized structural and material design to prevent heat dissipation into the air [23]. Continued advances in material selection and system integration remain essential to achieving thermo-haptic interfaces that are efficient, responsive, and skin-compatible, which is key to enhancing the immersive user experience.

**Table 3.** Comparative summary of mainstream wearable thermo-haptic systems

Types	Materials	Dimensions	Response time (temperature change)
Joule-heated [60–62]	Laser-induced graphene [60], Cu [61], eutectic gallium–indium [62]	Heating area: 5,221 mm^2^ [61], thickness: ≤0.35 mm [62]	<60 s (Δ32.2 °C) [60], <7 s (Δ13 °C) [61], ~75.48 s (from room temperature to 83.24 °C) [62]
Thermoelectric [14,69]	Bismuth telluride	Heating area: 729 mm^2^ [14], thickness: NR	3.52 s (heating mode, Δ*T* = 15 K) [14], 4.48 s (cooling mode, Δ*T* = 15 K) [14], <0.5 s (Δ10 °C) [69]

NR, not reported.

## Mechanical Interfaces for Tactile Feedback

### Pneumatic- and hydraulic-enabled wearable haptics

Mechanical stimulation plays a central role in haptic perception, with skin deformation providing critical cues in response to external forces [71]. While point-based actuators often struggle to replicate complex haptic interactions, pneumatic and hydraulic systems offer an effective route for delivering large, tunable forces to the skin by modulating internal fluid pressures [63]. These fluid-driven soft actuators conform to the body and dynamically adjust pressure and frequency to activate cutaneous mechanoreceptors. The resulting skin displacements generate lifelike tactile sensations, closely mimicking real-world physical interactions [72,73].

Viscous and inertial effects fundamentally limit the temporal response of fluidic actuators. For laminar flow in microchannels, the characteristic response time *τ* is dominated by the viscous diffusion time scale [74]:

$$\tau ∼\frac{L^{2}}{\nu }$$ (1)

where *ν* = *μ*/*ρ* is the kinematic viscosity, *μ* is the dynamic viscosity, *ρ* is the fluid density, and *L* is the characteristic channel length. This quadratic dependence explains why macroscopic systems exhibit latencies greater than 100 ms, while microscale hydraulics (e.g., hydraulically amplified electrostatic taxel [HAXEL] [18]) achieve sub-10-ms response. For compressible pneumatic systems, an additional acoustic time scale *τ* ∼ *L*/*c* (where *c* is the sound speed) must be considered [75]. Water (*ν* ≈ 1 mm^2^/s) responds faster than silicone oils (*ν* ≈ 50 to 100 mm^2^/s), but it requires higher pressures to overcome the microchannel surface tension.

**Fig. 4.**
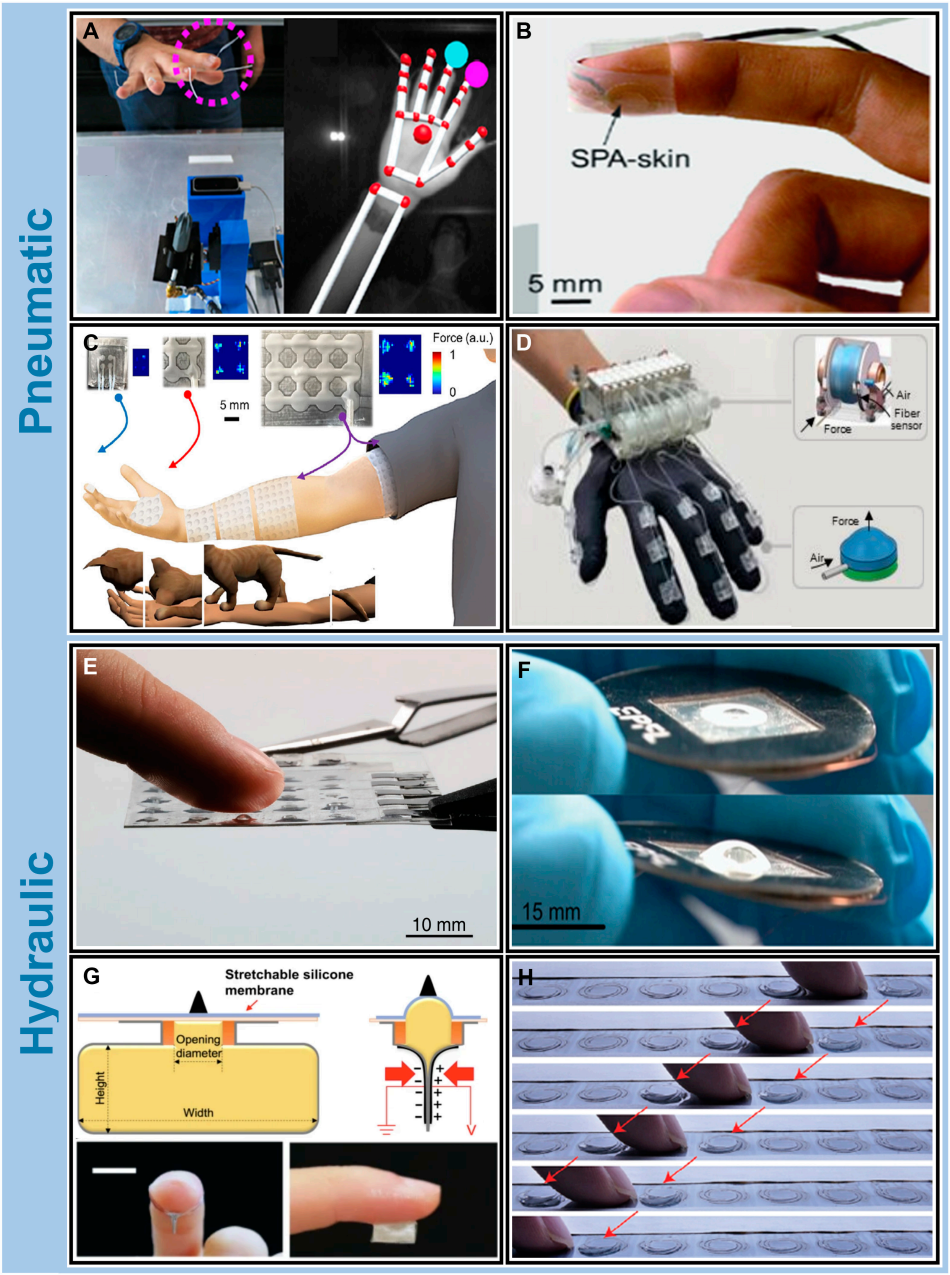
Pneumatic and hydraulic haptic wearable systems. Representative platforms for fluidic-based haptic feedback. (A) Pneumatic interface enabling independent tactile stimulation of the middle and index fingertips via directed air jets. Copyright 2021, Wiley [79]. (B) Bimodal feedback system integrating soft pneumatic actuator skin (SPA-skin) with moldable substrates to replicate texture and shape perception. Copyright 2021, Wiley [80]. (C) Wearable pneumatic haptic system using pixelated and patch-type airbag arrays to deliver localized feedback to fingertips and forearms in interactive environments. Copyright 2024, Cell Press [81]. (D) HaptGlove, an unconstrained pneumatic glove combining distributed air chambers with optical sensors for synchronized skin indentation and joint torque rendering in virtual reality. Copyright 2023, Wiley [82]. (E) Hydraulically amplified electrostatic taxel (HAXEL) using electrostatic mechanisms to achieve high energy density and rapid response without external compressors. Copyright 2020, Wiley [83]. (F) Zipper-effect-based HAXEL array with internal hydraulic amplification to enable site-specific, high-force tactile feedback in soft sticker formats. Copyright 2023, Wiley-VCH [18]. (G) Vertical, stretchable-pressure-amplified electrostatic actuator incorporating fluidic necking structures to enhance conversion efficiency for high-resolution haptic rendering. Copyright 2023, Springer Nature [84]. (H) Ultrathin hydraulically actuated tactile pixel array forming reconfigurable pressable buttons with large out-of-plane displacement and low power consumption for interactive surface control. Copyright 2023, Wiley [85]. Copyright as indicated; all reproduced with permission.

Pneumatic actuators regulate airflow to control mechanical deformation, enabling diverse tactile outputs such as pressure, vibration, and texture cues [76–78]. Christou et al. [79] developed a pseudo-holographic display integrated with a pneumatic haptic feedback module that delivers haptic cues through compressed air jets targeting the user’s hand (Fig. 4A). The system combines visualization, gesture recognition, and pneumatic actuation to generate directional force feedback up to ~6 N. Sonar et al. [80] introduced a soft pneumatic actuator skin (SPA-skin) capable of closed-loop feedback with a maximum frequency of 100 Hz and a blocked force of 1 N at 50 kPa. They later extended this concept by integrating their SPA-skin with a deformable substrate (e.g., playdough) to create a bimodal haptic system (Fig. 4B), where actuator pressure and substrate compliance and plasticity together enabled feedback modes such as roughness and shape sensing. Wang et al. [81] designed a wearable pneumatic interface composed of pixelated and patch-based capsule arrays tailored for fingertips and forearms, respectively (Fig. 4C). By tuning capsule size and spacing, the system matched spatial receptor distributions across body regions. Controlled air injection deforms individual capsules to stimulate localized skin regions, achieving programmable feedback across a pressure range of 20 to 100 kPa. In a related approach, Qi et al. [82] developed HaptGlove, a wearable pneumatic glove integrating distributed actuators with stretchable fiber sensors for precise skin indentation control (Fig. 4D). By dynamically modulating air pressure and excitation frequency, the glove delivers synchronized skin and joint feedback in virtual environments, improving immersion and tactile realism.

Hydraulic actuators generate mechanical deformation through liquid-mediated pressure changes, offering high force density and low response latency. Leroy et al. [83] introduced a multimode HAXEL that delivers compact, compressor-free actuation with rapid feedback and elevated energy density (Fig. 4E). Building on this design, the team later developed a zipper-driven HAXEL system incorporating a hydraulic amplification mechanism (Fig. 4F) [18]. In this configuration, localized actuator displacement, induced by the zipper mechanism, drives fluid movement, generating out-of-plane expansion with force outputs ranging from 100 to 800 mN and displacements between 100 and 850 μm.

At the microstructural level, Chen et al. [84] designed a stretchable, pressure-amplified electrostatic actuator based on a vertical architecture combining a flexible electrode and a fluidic amplification chamber (Fig. 4G). Upon voltage application, electrostatic attraction induces fluid motion, while a necked geometry enhances energy conversion. The device achieves a displacement of ~500 μm under 1 MPa and, when paired with a porous-material pressure sensor, enables a closed-loop HMI (CL-HMI) capable of tactile texture and shape recognition. Firouzeh et al. [85] created a thin (500 μm) hydraulically amplified electrostatic actuator capable of reconfigurable out-of-plane motion (Fig. 4H). High-voltage electrostatic forces cause a zippered membrane to collapse onto the substrate, displacing liquid into a domed elastomer cap and generating a 1.5-mm elevation, functionally mimicking the mechanism of a mechanical button. Each pixel consumes only 6 mW, supporting scalable, low-power implementation of high-fidelity tactile surfaces.

Although pneumatic and hydraulic actuators provide robust haptic and kinesthetic feedback, they are often constrained by substantial weight and bulky designs. Generating internal pressure typically requires external compressors, tubing, and fluidic components, which substantially limit system portability and integration. While these actuators offer wide-area coverage and high force output, their performance is hindered by slow response times due to the inertia of fluid movement, an inherent limitation relative to other haptic modalities [63]. Future advances may come from the development of lightweight structural materials and highly integrated microfluidic architectures that eliminate the need for bulky external systems. In parallel, intelligent control algorithms that optimize fluid dynamics could reduce latency and enhance responsiveness, broadening the applicability of pneumatic and hydraulic haptic interfaces in mobile and wearable platforms.

### Electromagnetic-enabled wearable haptics

Electromagnetic-enabled haptic wearable systems are a mature and versatile class of wearable feedback technologies. These devices operate on the principle of electromagnetic induction: When a time-varying current flows through a coil, it generates a magnetic field that exerts a Lorentz force on an embedded magnet. The resulting displacement deforms the skin and elicits mechanical stimulation, giving rise to a perceived haptic sensation [60,86]. Compared to other modalities, electromagnetic actuators offer several advantages, including fast response times, low power consumption, and compact form factors. Their ability to operate wirelessly without bulky pressurization hardware and other actuation interfaces further expands their integration potential in conformal wearable platforms [87,88]. Common implementations include eccentric rotating mass (ERM) and voice coil actuators, both adopted in emerging wearable systems [5].

**Fig. 5.**
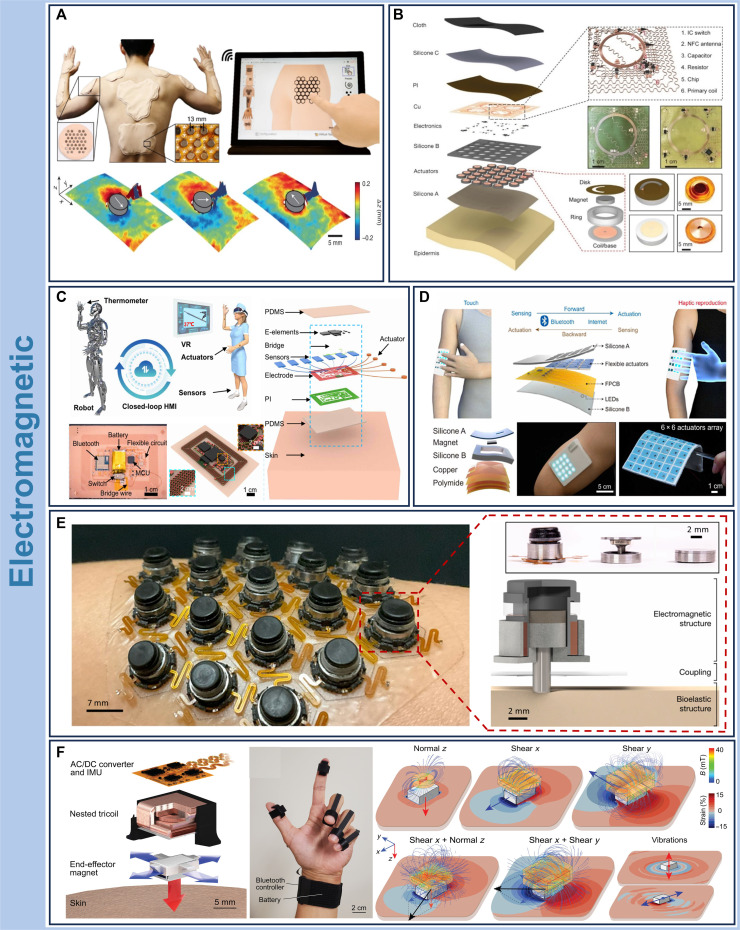
Electromagnetic haptic wearable systems. Representative platforms for electromagnetic-based haptic feedback. (A) High-density hexagonally tiled actuator array integrating up to 147 eccentric rotating mass (ERM) units for distributed haptic feedback across multiple body regions. Copyright 2022, Springer Nature [19]. (B) Exploded schematic of a large-scale, wireless electromagnetic actuator array for flexible skin interfaces. IC, integrated circuit. Copyright 2019, Springer Nature [22]. (C) Closed-loop electronic skin (e-skin) enabling full-body motion capture and real-time haptic interaction in virtual environments. MCU, microcontroller unit. Copyright 2022, AAAS [25]. (D) Self-sensing, soft electromagnetic actuator array forming a reproducible and conformal haptic e-skin platform. LEDs, light-emitting diodes. Copyright 2022, AAAS [20]. (E) Bistable haptic interface leveraging skin elasticity as an energy storage medium to enable multimodal tactile stimulation, including pressing, vibration, and torsional feedback. Copyright 2024, Springer Nature [93]. (F) A full freedom-of-motion (FOM) actuator system delivering programmable normal, shear, and torsional forces for immersive haptic feedback and spatial navigation. IMU, inertial measurement unit. Copyright 2025, AAAS [94]. Copyright as indicated; all reproduced with permission.

ERM actuators are widely adopted in wearable haptic devices due to their structural simplicity, low cost, and ease of integration [89]. ERMs operate by driving a motor shaft via electromagnetic induction, to which an asymmetrically mounted mass is attached. As the shaft rotates, centrifugal forces generated by the unbalanced mass induce periodic vibrations [90]. Owing to their Euler’s disk-like rotational dynamics, ERM also produce lateral shear forces to the skin, which, when combined with vertical vibrations, elicit robust tactile sensations even in regions with sparse mechanoreceptor density. Jung et al. [19] developed a high-resolution, full-body haptic interface based on ERM technology, capable of delivering complex feedback patterns across large skin areas (Fig. 5A). The system uses a dense actuator array to enable multimodal stimulation across different body regions and features a wireless graphical user interface for real-time spatial control of vibration contours. Operating at a peak power of 158 mW, the actuators achieve vertical and lateral displacements of 0.23 and 0.86 mm, respectively, demonstrating the pronounced vibratory performance of skin-integrated ERM systems.

Voice coil actuators, which rely on electromagnetic forces to generate motion, are widely used in applications requiring precise vibration and displacement control [22,86]. These actuators offer efficient and precise tunable haptic feedback across diverse platforms [91,92]. Yu et al. [22] introduced the first wireless, battery-free voice-coil-based actuator array designed for integration into soft, flexible epidermal electronics (Fig. 5B). The system comprises 32 independently operated actuators, wirelessly linked by near-field communication (NFC) protocols and embedded in a stretchable circuit layout. Each millimeter-scale actuator produces localized mechanical vibration under wireless control, enabling spatially resolved haptic feedback. Compared to commercially available alternatives, which typically consume over 100 mW per unit, the optimized actuators operate at only ~1.75 mW while delivering a perceptible force of 135 mN to the skin, sufficient to elicit distinct haptic sensations at the fingertips and palm.

Liu et al. [25] developed a wireless, CL-HMI system based on voice coil actuators, integrating both sensing and feedback components for remote robotic teleoperation (Fig. 5C). Mounted on the skin as an electronic epidermis, the system captures full-body motion in real time and transmits it wirelessly to a remote robot, while simultaneously delivering haptic feedback to the user. In a demonstration involving remote nucleic acid sampling, medical personnel wore flexible electronic skin (e-skin) equipped with bend sensors to record motion commands. These signals guided robotic manipulation, and haptic feedback was relayed to the user to enhance control precision. In a separate study, Li et al. [20] reported a CL-HMI platform comprising a soft e-skin embedded with an array of miniaturized electromagnetic actuators (Fig. 5D). The actuator core features a trilayer flexible copper coil fabricated on an 18-μm-thick polyimide (PI) film, encapsulated within a polydimethylsiloxane (PDMS) matrix. The PDMS substrate offers superior mechanical flexibility, skin conformity, and biocompatibility, while encapsulated magnets enable mechanical actuation without compromising softness. This soft system minimizes user discomfort and supports bidirectional haptic communication. In remote education scenarios, the CL-HMI functions as a tactile walkie-talkie, allowing instructors to transmit physical cues through e-skin, while students receive and actively respond with touch-based feedback. The integration of soft materials and wireless electronics in these systems provides a versatile platform for precise, real-time control and haptic feedback across healthcare, education, and teleoperation applications.

Expanding the versatility of electromagnetic haptic systems, researchers have developed platforms capable of delivering multimodal mechanical stimuli with enhanced energy efficiency. Figure 5E shows a wireless, low-power electromagnetic skin interface that leverages the skin’s inherent elastic recovery to generate dynamic and static haptic feedback [93]. The device improves energy efficiency by recovering elastic energy stored in the skin, enabling displacements exceeding 2 mm and forces up to 1.4 N. Arrays of these bistable transducers are integrated on a flexible, skin-conformable substrate and can independently deliver both dynamic vibratory and static indentation stimuli. Vibrotactile actuation is driven by applying an AC at subtransition amplitude, which vibrates the armature with tunable frequency and amplitude to elicit distinct tactile perceptions. Static indentation is achieved through bistable operation via pulsed DC: transitioning from the compressed state to the relaxed state by driving the coil with opposite polarity to the permanent magnet and from the relaxed to the compressed state by driving the coil in alignment with the magnet, thereby maintaining localized skin deformation for sustained mechanical feedback. By exploiting kirigami structures that convert normal forces into tangential forces and through independent spatiotemporal control of indentation and vibration, the system also supports the perception of directional shear and textured cues. Participants reported significantly enhanced perception intensity with torsional angles between 5° and 15°. This bioinspired haptic system has shown considerable promise in assisting individuals with sensory impairments, supporting applications in tactile navigation, balance assistance, and gait stabilization/support.

Figure 5F presents a full freedom-of-motion (FOM) actuator capable of delivering multidirectional, superimposed mechanical stimuli to the skin surface [94]. The device integrates 3 orthogonally arranged electromagnetic coils to generate normal, shear, and torsional forces, within the perceptible range of human skin. When deployed as an array, FOM actuators enable spatially distributed and temporally programmable haptic feedback, supporting the simulation of complex tactile experiences such as realistic texture rendering and dynamic interaction cues. This capability lays the groundwork for high-bit-rate haptic communication and immersive virtual environments. Potential applications include multisensory interaction in extended reality, tactile navigation aids for individuals with visual impairments, and even somatosensory substitution for musical perception. Furthermore, the device architecture enables targeted stimulation of distinct mechanoreceptor populations, or combinations thereof, across large skin regions, offering a powerful platform for advanced somatosensory research and feedback systems.

Electromagnetic-enabled haptic wearable devices offer distinct advantages, including miniaturization, skin-conformal integration into thin and flexible platforms, well-established power harvesting/transmission strategies, and modular design for assembling large-area actuator arrays across diverse body regions. These features enable high spatiotemporal resolution and localized control of haptic stimuli. However, the intrinsic rigidity of embedded magnets introduces a mechanical mismatch with soft biological tissue, often resulting in discomfort during prolonged use. This limitation constrains their long-term applicability in wearable haptic systems [5,95]. Future efforts should focus on reducing power consumption and form factors and enhancing mechanical compliance with the skin to improve wearability and broaden their utility across interactive, medical, and immersive environments.

### Piezoelectric-enabled wearable haptics

The piezoelectric effect describes the ability of certain materials to generate surface charges in response to mechanical stress or, conversely, to undergo mechanical deformation when exposed to an electric field. This phenomenon arises from the spatial rearrangement of electric dipoles within the material. Under mechanical loading, the symmetry of the dipole configuration is disrupted, resulting in charge separation and surface polarization. Conversely, the application of an electric field reorients the dipoles, producing macroscopic strain. This bidirectional energy conversion between mechanical and electrical domains enables the use of piezoelectric materials in a wide range of applications. Today, piezoelectric haptic wearable devices are broadly deployed across sensing, actuation, and energy harvesting systems [96,97].

**Fig. 6.**
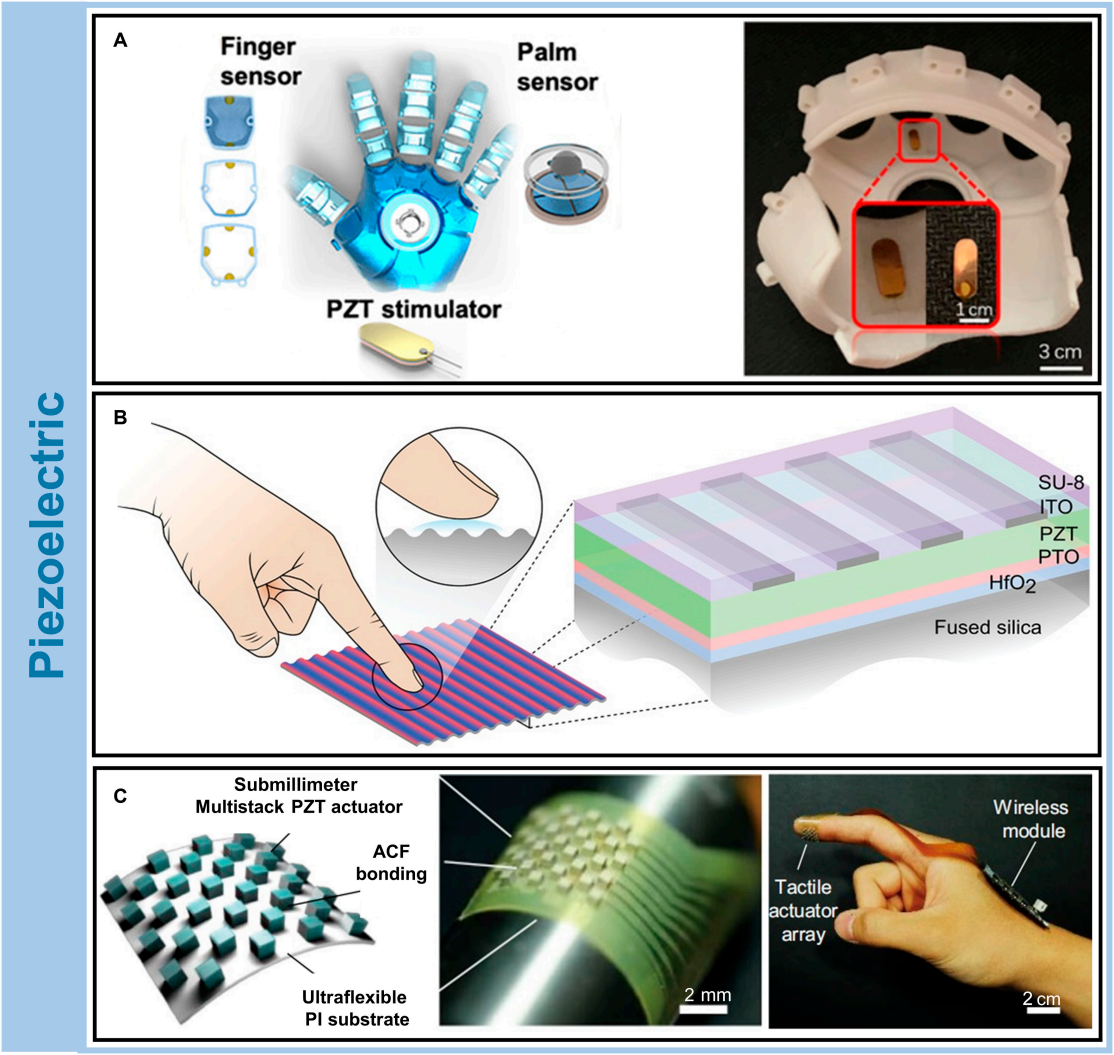
Piezoelectric-enabled haptic wearable systems. Representative platforms for piezoelectric-based haptic feedback. (A) Schematic of a haptic-feedback smart glove (left) and photograph of the integrated piezoelectric actuator (right). PZT, lead zirconate titanate. Copyright 2020, AAAS [98]. (B) Illustration of a friction modulation mechanism via acoustic vibration between a fingertip and device surface (left) and cross-sectional schematic of the fully transparent multilayer stack (right). Copyright 2020, Wiley [99]. (C) Three-dimensional schematic of a flexible submillimeter piezoelectric actuator array (left), image of the ultraflexible array (middle), and demonstration of the wireless system integrated on the hand for haptic rendering (right). ACF, anisotropic conductive film. Copyright 2022, Springer Nature [100]. Copyright as indicated; all reproduced with permission.

Building on these fundamental properties, researchers have engineered wearable piezoelectric systems capable of delivering precise, real-time haptic feedback in virtual environments. Zhu et al. [98] developed an intelligent glove integrating piezoelectric haptic feedback for next-generation human–machine interaction in VR/AR environments (Fig. 6A). The glove features a piezoelectric actuator with a rapid response time of 30 to 40 ms, enabling near-instantaneous vibration feedback. The actuator supports high-precision control and is well suited for generating complex haptic effects, particularly in friction modulation at ultrasonic frequencies. Output performance is tuned by altering triboelectric interaction conditions, such as contact area and separation velocity. In addition, vibration intensity can be modulated by adjusting the input voltage from 6 to 12 V. The system supports a peak feedback force of 3.5 N and detects forces ranging from 0.1 to 3.5 N, allowing incremental tactile responses. Operating at an optimal frequency of 270 Hz, the device maintains low energy consumption while delivering strong mechanical feedback. By integrating triboelectric sensors, piezoelectric actuators, and real-time voltage control, the glove enables efficient, low-cost, and multidimensional haptic interaction, greatly enhancing user experience in immersive digital environments.

To further expand the functionality of piezoelectric systems, recent work has focused on enhancing transparency and integration with optical surfaces. Glinsek et al. [99] reported the design and characterization of a fully transparent, tribo-modulated haptic device based on piezoelectric thin films (Fig. 6B). High-frequency vibrations generated by the piezoelectric layer produce a thin air film between the fingertip and the device surface, effectively reducing friction. The device operates through a multilayer structure engineered for mechanical performance and optical clarity. A transparent substrate provides mechanical support, while a hafnium dioxide (HfO_2_) buffer layer prevents interfacial reactions between the piezoelectric film and the substrate. An interdigitated indium tin oxide (ITO) electrode design reduces capacitance and enhances transparency, and an insulating layer ensures electrical safety. The device provides obvious haptic feedback, achieving a characterized out-of-plane displacement reaches almost 3 μm at 150 V.

In parallel, flexible form factors have enabled conformal piezoelectric interfaces capable of real-time feedback across curved and dynamic skin surfaces. Jin et al. [100] developed an ultraflexible piezoelectric haptic interface with a spatial resolution (1.8-mm pitch) for tele-haptic communication on human skin (Fig. 6C). The system integrates submillimeter-scale piezoceramic actuators and dual-mechanism sensors onto a checkerboard-patterned array spanning approximately 1 cm^2^. The array is fabricated on an ultraflexible substrate that conforms naturally to the curvature of the fingertip. In remote haptic transmission experiments, the interface successfully conveyed shape and vibration cues with high fidelity and low latency (<1.55 ms), underscoring the responsiveness of piezoelectric systems. Despite these design advantages, the actuator’s maximum displacement of 1.3 μm constrains its ability to replicate strong tactile events, such as impacts or collisions, thereby limiting its utility in high-intensity haptic scenarios.

Piezoelectric-enabled modalities remain a compelling choice for haptic actuators due to its rapid response times and compatibility with slim, lightweight form factors [101,102]. Nonetheless, several limitations continue to hinder broader adoption. The usable bandwidth is typically narrow with a nonuniform frequency response, and this can be mitigated by multifrequency synthesis or beat-based rendering with drive compensation [103–105]. To address the issue of insufficient mechanical flexibility, the integration of piezoelectric functionality with flexible electronics technology has been achieved, enabling conformal integration with curved surfaces and multimodal interaction [61,106]. Low-frequency displacement is often insufficient, and this can be improved using resonant designs or displacement-amplifying structures [104,107]. Looking ahead, advances in materials and actuator design may unlock new applications for piezoelectric haptics in domains ranging from teleoperated robotics and intelligent prosthetics to immersive virtual environments.

Table 4 presents a comparative evaluation of wearable mechanical haptic systems. ERM and voice coil actuators typically feature dimensions on the millimeter scale and unit masses on the order of grams (approximately 1 g) [108]. With their low power consumption and low drive-voltage requirements, they are well suited for low-power, wireless, large-area skin-integrated systems [19,22]. Owing to their compact form factor [100], piezoelectric actuators are commonly used for high-resolution tactile rendering. Nevertheless, they generally require higher drive voltages and have output displacements of less than 35 μm [98–100,109]. Pneumatic and hydraulic systems can deliver higher force outputs and are effective at simulating sustained skin-pressure sensations. Hydraulic actuators, for example, are reported to generate normal forces on the order of several newtons [110]. Although the individual actuators are compact, the requirement for external compressors or pumps results in substantially larger system volume and weight, as well as higher integration complexity. For example, a pneumatic vest may use up to 26 independent airbags, further compromising wear comfort and portability [111].

**Table 4.** Comparative evaluation of wearable mechanical haptic systems

Type	Size	Weight	Power consumption	Response time	Vibration	Normal displacement	Tangential displacement
Voice coil actuators [22,24,25]	Actuator radius: 2.5–9 mm, actuator thickness: >1.45 mm	NR	>1.4 mW	<3 ms	60–300 Hz	~0.20–1.55 mm	NR
Eccentric rotating mass [19,108]	Actuator radius: >5 mm, actuator thickness: NR	1.29 g [108]	12.7–158 mW	~20 ms	70–200 Hz	350 μm	860 μm
Piezoelectric [98,99,109]	Actuator thickness: <2.3 mm	NR	~170 mW	1.55–40 ms	50–500 Hz	1.3–35 μm	NR
Pneumatic [79,80,82,111]	NR	5 lbs [111]	NR	≤0.8 s	0–100 Hz	NR	NR
Hydraulic [18,83–85,110]	NR	<8 g [110]	NR	NR	0–500 Hz	5 mm [110]	NR

NR, not reported.

Selection of actuators for tactile feedback, informed by the comparative analyses in Tables 1 and 4, depends on matching actuator output to receptor-specific detection thresholds. Within the frequency range of 5 to 50 Hz, Meissner and Merkel receptors are predominantly responsive, requiring a threshold skin deformation exceeding approximately 10 μm [29,30]. Some piezoelectric actuators achieve displacements of up to 35 μm, meeting or exceeding the displacement required for low-frequency mechanoreceptor activation, thereby enabling reliable rendering of tactile cues such as taps, slips, and surface roughness [109]. In contrast, another design provides a displacement of only about 1.3 μm, which is insufficient to activate fast-adapting type I receptors at low frequencies but is well suited for higher-frequency operation, such as in the 150- to 300-Hz range, where such displacements suffice to stimulate Pacinian corpuscles [100]. ERM and voice coil actuators generate displacements on the order of several hundred micrometers [19], and fluid systems can readily produce millimeter-scale indentations [110]. In haptic feedback systems, mechanical stimulation offers notable advantages, including naturalistic interaction and high safety, and is particularly well suited for conveying physical cues such as texture, vibration, and pressing/indentation [86].

## Multimodal-Enabled Wearable Haptics

Multimodal wearable haptic systems integrate multiple physical stimuli to enhance perceptual realism and immersion in interactive applications. By enabling mechanical, thermal, and electrical cutaneous channels simultaneously, these systems reproduce complex tactile experiences that surpass the capabilities of single-modality vibration. Early work by Yem and Kajimoto [112] demonstrated a fingertip interface that combined mechanical actuation with electrotactile stimulation for virtual interaction, laying the groundwork for subsequent multimodal designs. Subsequent efforts focused on integrating pressure and thermal feedback into wearable form factors. Cai et al. [113] developed a pneumatic glove that couples air-pressure cues with actively controlled heating and cooling, enabling material identification in virtual reality tasks. Zhang and Sra [114] introduced PneuMod, a modular on-body system in which reconfigurable units deliver localized pneumatic pressure and thermoelectric thermal feedback, demonstrating that modularity enables deployment across multiple anatomical locations and usage scenarios. Recent advances emphasize miniaturization and seamless skin integration. Huang et al. [23] presented a skin-integrated multimodal haptic interface that integrates thermoelectric, electrotactile, and electromagnetic actuators on an ultrathin, flexible substrate, enabling wireless operation with spatially resolved tactile output. This platform exemplifies the codesign of multiple modalities within soft electronic architectures, enabling fine spatial and temporal control for extended reality and teleoperation.

Although multimodal tactile systems can provide richer, more realistic tactile experiences, cross-modal interference may occur between different stimuli. For example, when thermal, electrical, and mechanical modules operate on the same skin site, one stimulus can inadvertently alter the operating conditions of another [115]. Electrothermal coupling arises because changes in skin temperature can alter skin–electrode impedance and perception thresholds, causing the same electrotactile waveform to feel different over time or across body sites [116]. On the hardware side, adaptive compensation can be achieved by dynamically monitoring impedance variations and adjusting electrical stimulation parameters in real time [36]. Similarly, electromechanical coupling can occur when electrical and mechanical stimuli (such as vibration or indentation) are applied simultaneously. In this case, the mechanical stimulus may cause partial electrode detachment, disrupting skin–electrode contact, altering the interface impedance, and ultimately resulting in unstable perceived intensity of the electrical stimulation [36,37]. Advanced electrode materials that maintain robust contact are critical for suppressing interference from mechanical stimuli, thereby minimizing impedance fluctuations caused by poor adhesion or detachment [40,45]. Localized heating may induce thermomechanical coupling by softening the skin and tissues, altering mechanical contact properties at the skin–device interface [117]. Consequently, the same mechanical input can yield different perceptual outcomes. A practical way to mitigate thermomechanical coupling is to physically separate thermal and mechanical paths in the device layout, for instance, by placing heating elements away from primary mechanical contact regions, thereby minimizing thermal influence on actuation output [23]. In addition, dynamic power allocation can further reduce interference by prioritizing modalities and scheduling actuator drive signals to avoid overlapping high-power events, thereby improving multimodal stability and perceptual coherence [63].

Future research should treat cross-modal interference as a primary challenge [81]. At the same time, it is crucial to improve energy efficiency and reduce size without compromising output performance [63]. In addition, advancing low-power thermal management, enabling efficient thermal–electrical–mechanical energy conversion and distribution, and implementing smart interfaces capable of real-time physiological sensing and adaptive stimulation adjustment remain critical research priorities [86]. As these challenges are addressed, multimodal wearable haptic systems are poised to enable immersive tactile interaction across virtual and augmented reality, teleoperation, training, and assistive technologies.

## Discussion

Table 5 summarizes the capabilities and key characteristics of mainstream haptic modalities. In electrical stimulation, monophasic pulses use simple, low-power drivers and evoke tingling or pressure sensations, along with static spatial cues, but they raise net charge safety concerns and are highly sensitive to skin–electrode impedance [4]. Biphasic pulses provide charge-balanced operation and more perceptually stable directional or continuous cues, although they require higher-compliance drivers and more complex circuitry [13]. Amplitude-modulated high-frequency AC stimulation supports vibration, texture rendering, and high spatial resolution, but it requires careful waveform tuning and increased circuit complexity to avoid paresthesia [41]. Thermal stimulation strategies exhibit distinct performance trade-offs. Joule-heating approaches produce warm sensations with simple device architectures, low cost, and straightforward integration [63]. However, they provide heating only, respond slowly due to thermal inertia, risk localized overheating without feedback control, and lack active cooling [86]. Thermoelectric devices support both warming and cooling with precise bidirectional control and closed-loop operation, but they suffer from low energy efficiency, challenging thermal management, and increased system mass or volume [118]. Mechanical stimulation modalities exhibit distinct performance criteria that reflect their underlying actuation mechanisms [13,119]. Pneumatic actuation generates indentation, protrusions, normal force cues, and spatial patterns and offers a long stroke, high compliance, user comfort, and intrinsic safety [63]. However, reliance on compressors and tubing increases the system's footprint and complexity [115]. Hydraulic actuators deliver high force density, long stroke, and stable load holding, enabling sustained pressure cues and deep indentation [86]. These advantages come at the cost of heavy peripheral components, a risk of fluid leakage, slow response times, and increased maintenance requirements [81].

**Table 5.** Concise summary and comparison across mainstream haptic modalities

Modalities	Type	Advantages	Disadvantage	Typical applications	Ref.
Electrical stimulation	Monophasic pulses	Simple, low cost	Lower safety	Electrotactile feedback	[34,35,40,45,48]
Biphasic pulses	Higher safety	Complex	Navigation	[49–52]
Amplitude-modulated, high-frequency AC	Higher safety, high spatial resolution	Complex	Texture rendering	[41]
Thermal stimulation	Joule heater	Simple structure, low cost	Heating-only, high latency	Cutaneous warming feedback	[60,62]
Thermoelectric	Bidirectional control	Low efficiency, added volume	Bidirectional thermal feedback	[14,61,69,118]
Mechanical stimulation	Pneumatic/hydraulic	High force output	High latency, bulkiness	Force/pressure feedback	[18,82,110]
Piezoelectric	High precision	High-voltage drive	High-frequency vibrotactile texture	[109,119]
Electromagnetic	Low latency, low power	Narrow bandwidth	Vibrotactile feedback	[19,22,108]

Beyond these modality-specific capabilities and constraints, energy efficiency and response speed are also key metrics for comparison. From an energy efficiency perspective, electromagnetic and electrostatic actuators operate at very low power and exhibit millisecond-scale response times, making them well suited for battery-powered wearable applications [63]. In contrast, thermal devices, including joule heaters and thermoelectric systems, as well as fluid-based platforms such as pneumatic, hydraulic, and microfluidic actuators, consume more power and respond more slowly [13]. These characteristics reduce overall energy efficiency and limit use to scenarios where power availability is less constrained [86].

While energy efficiency and response speed are critical for a modality’s technical feasibility, its ultimate acceptance and utility in real-world scenarios depend on user-centric factors, primarily comfort and wearability. These factors are critical for users and practical deployment, yet difficult to quantify with a single metric [120], so we discuss them qualitatively. Pneumatic and hydraulic haptic systems typically rely on external pumps, valves, power supplies, and tubing, which often restrict mobility or increase the wearing burden [63]. In contrast, although increasing the number of ERM and voice coil actuators in large-scale arrays increases overall weight, their ability to enable fully wireless, skin-integrated designs can greatly enhance wearing comfort [71,86]. Joule-heating devices can be thin and wearable, but sustained heating may feel stuffy or cause burning sensations, and they often require additional heat-spreading or cooling structures [81]. Thermoelectric systems can provide cooling, but they are often bulky and power-intensive [13]. Electrotactile feedback can be lightweight, but comfort depends strongly on a stable skin–electrode interface [63]. Changes in impedance caused by sweating, temperature variation, and motion can lead to intensity drift [4]. Therefore, improved interface materials and adaptive driving strategies are needed to balance perceptual strength and comfort [45].

Alongside technical and user-centric considerations, the commercial scalability of a haptic modality is largely determined by its cost structure, which varies considerably across different actuation principles. ERM and voice coil actuators are generally low cost, with a single ERM typically priced at US$2.88 [19]. The overall system cost after integration depends largely on the choice of control and wireless modules, including their chips and associated electronics. In comparison, compact piezoelectric actuators have a higher unit cost of approximately US$19.63 [119]. Pneumatic and hydraulic actuators shift the cost from the actuator itself to supporting hardware. For example, while a hydraulic tactile element may cost less than €1 in materials [110], the total system expense is driven mainly by pumps, compressors, valves, reservoirs, and tubing. Among thermo-haptic systems, joule-heating devices typically have low unit costs, but overall system cost increases substantially because of required heat-insulation structures, thermal-dissipation design, and safety-limiting circuitry [121]. In contrast, thermoelectric solutions require integrated thermoelectric components and corresponding heat management modules, which often lead to higher overall material costs[122]. For electro-haptic systems, the electrodes themselves are inexpensive [37]. However, dedicated driver circuits are often required to precisely control stimulation waveforms and ensure safety and effectiveness, and these circuits typically account for most of the added cost [37,41]. In summary, the total cost of a haptic system is driven not only by the actuator unit price but also by supporting drive electronics, packaging and integration, and safety and reliability design [115]. When scaled to multichannel or large-area arrays, costs typically increase substantially with actuator count and control complexity [37,115].

In addition to cost considerations, the feasibility of a reliable wearable product depends primarily on addressing barriers to practical application. The practical application of wearable haptic systems is currently limited mainly by manufacturing scalability and long-term biocompatibility [45,54,89,122]. Many high-performance designs rely on complex fabrication processes, where the manual precision required for prototyping is difficult to reconcile with the efficiency and consistency demanded in mass production [89]. Long-term skin-conformal wear also faces challenges from sweat, friction, temperature, and humidity fluctuations, as well as repeated donning and doffing [45,122,123]. Common risks include adhesive failure, encapsulation aging, skin irritation, and similar issues. Feasible countermeasures include prioritizing commercially available components and mature processes to reduce assembly and integration complexity, thereby lowering overall cost and improving practical feasibility [89]. It is also important to adopt more skin-friendly, breathable materials and more reliable encapsulation, define clear safety thresholds, and verify stability through long-term wear tests [45,122,123].

The selection of an appropriate haptic feedback strategy depends on the specific needs of the target application, including energy efficiency, wearing comfort, cost, and practical implementation barriers. Future advances should focus on tighter system integration, higher energy efficiency, and continued miniaturization, especially for multimodal architectures designed for mobile and wearable platforms.

## Conclusion and Future Prospects

This review highlights recent progress in wearable haptic systems, offering a systematic evaluation of recent key technologies from the perspectives of structural design, actuation mechanisms, and advanced materials. Focusing on 3 primary modalities, electrical, thermal, and mechanical stimulation (including pneumatic/hydraulic actuators, voice coil motors, ERMs, and piezoelectric actuators), we summarize their operational principles, strengths, and limitations. Through representative wearable applications spanning medical rehabilitation, immersive entertainment, educational training, and industrial automation, we analyze how each approach uniquely addresses the demands of different user environments.

Electrical stimulation systems are characterized by simple architectures and mature fabrication protocols, enabling integration of flexible and stretchable electrodes with high spatial resolution [33,37,41,46,116]. However, fine-tuning stimulation parameters remains a challenge, especially when accounting for individual variability in tactile sensitivity [31,32,36,116]. Thermal feedback devices, while less demanding in spatial resolution, can convincingly replicate temperature changes to enhance immersion [14,53,61]. However, their performance is often limited by the slow dynamics of heat transfer and a lack of high-efficiency, flexible thermoelectric materials [53,64,65]. Mechanical actuators deliver high-force output and intuitive, skin-deforming sensations that closely mimic real-world interactions [16,22,86,94]. Nonetheless, their miniaturization and integration into compact wearable formats remain technically demanding due to the complexity of their assembly and actuation requirements [24,71].

Achieving physiologically relevant tactile feedback will require more than miniaturization and material innovation; it will depend on deeper biological insight into mechanoreceptor function and how to transduce neural signals into programmable mechanical stimuli [29,32]. Future development of wearable haptic systems should prioritize lightweight, skin-integrated reconfigurable designs that incorporate ultrathin electrodes and advanced functional layers via scalable micro/nanofabrication techniques [22,23,45,89,96,101]. Integrating real-time sensing with closed-loop, intelligent control will allow dynamic and personalized modulation of stimulation profiles [11,20,35,84].

Multimodal haptic integration, which combines mechanical, thermal, and electrical stimuli, enables richer and more immersive tactile interactions than single-modality feedback [13,86]. However, the comparative analysis in this review indicates that simple colocation of modalities is insufficient [23]. Effective designs require holistic codesign strategies that mitigate energy coupling and cross-modal interference through dynamic power allocation, adaptive closed-loop control, and physical decoupling [81]. Long-term usability will hinge on advances in low-power, wireless power delivery systems and effective thermal management strategies to enable continuous, prolonged operation without compromising user comfort [63]. Innovations in soft, stretchable materials with tailored mechanical properties will further improve functionality, conformability, wearability, and biomechanical compatibility with complex anatomy, enabling new form factors and enhanced user comfort in dynamic environments.

Despite ongoing technological advances in wearable haptic systems, large-scale commercialization remains constrained by several nontechnical barriers [115,124,125]. Business models and market positioning remain poorly defined [124–126], with return on investment difficult to quantify for enterprise users, including industrial and medical stakeholders [124,125], and consumer adoption still maturing [126]. In parallel, the absence of unified industry standards and regulatory frameworks presents a systemic challenge [127–129]. Core elements such as data interfaces, communication protocols, and the encoding and safety of haptic feedback lack globally harmonized specifications [127,128], and in regulated domains such as healthcare, this gap is compounded by limited dedicated approval guidance for haptic devices, which prolongs development timelines and increases uncertainty in time to market [125,129]. Social acceptance and ethical considerations further constrain adoption [130,131], as continuous collection of physiological and behavioral data raises concerns regarding data privacy, ownership, and security [132,133], and the potential for haptic feedback to influence emotional or behavioral responses leaves the long-term psychological and societal effects of sustained use insufficiently understood [130,131].

In summary, wearable haptic systems are well positioned to profoundly reshape the landscape of digital interaction through experiences that rely on multiphysics stimuli to reproduce, fully or partially, lifelike interactions. As key technical challenges are addressed and new application domains emerge, these systems will play an increasingly critical role in enhancing sensory communication, enabling intuitive HMIs, and expanding the boundaries of immersive experiences.
